# Comparing 2-dimensional macular pigment optical density with objective and subjective perimetry and visual acuity in age-related macular degeneration

**DOI:** 10.1007/s00417-024-06437-6

**Published:** 2024-03-14

**Authors:** Bhim B. Rai, Faran Sabeti, Joshua P. van Kleef, Corinne F. Carle, Emilie M. F. Rohan, Rohan W. Essex, Richard C. Barry, Ted Maddess

**Affiliations:** 1 John Curtin School of Medical Research, Australian National University, Building 131, Garran Road, Canberra, ACT 2601 Australia; 2 Faculty of Health, School of Optometry, University of Canberra, Canberra, ACT Australia; 3 Department of Ophthalmology, Canberra Hospital, Canberra, ACT Australia; 4 Blink Eye Clinic, Canberra, ACT Australia

**Keywords:** AMD, Intermediate AMD, Macular pigment density, Multifocal, Objective perimetry, 2D-MPOD

## Abstract

**Purpose:**

To compare diagnostic power for different severities of age-related macular degeneration (AMD) of two-dimensional macular pigment optical densities (2D-MPOD) and spatially matched objective perimetry, with standard perimetry and best-corrected visual acuity (BCVA).

**Methods:**

The ObjectiveField Analyser (OFA) provided objective perimetry, and a Heidelberg Spectralis optical coherence tomography (OCT) measured 2D-MPOD in AMD patients, both completed twice over 0.99 ± 0.16 years. From each 2D-MPOD image, we extracted 20 regions/macula, matched to the 20 OFA stimuli/macula. For each region, we calculated 7 measures from the 2D-MPOD pixel values and correlated those with OFA sensitivities and delays. We quantified 2D-MPOD changes, the ability of 2D-MPOD and OFA to discriminate AMD stages, and the discriminatory power of Matrix perimetry and BCVA using percentage area under receiver operator characteristic plots (%AUROC).

**Results:**

In 58 eyes of 29 subjects (71.6 ± 6.3 years, 22 females), we found significant correlations between 2D-MPOD and OFA sensitivities for Age-Related Eye Disease Studies (AREDS)-3 and AREDS-4 severities. Delays showed significant correlations with AREDS-2. For AREDS-4, correlations extended across all eccentricities. Regression associated with the Bland–Altman plots showed significant changes in 2D-MPOD over the study period, *especially variability measures*. MPOD per-region medians discriminated AREDS-1 from AREDS-3 eyes at a %AUROC of 80.0 ± 6.3%, outperforming OFA, Matrix perimetry, and BCVA.

**Conclusions:**

MPOD changes correlated with central functional changes and significant correlations extended peripherally in later-stage AMD. Good diagnostic power for earlier-stage AMD and significant change over the study suggest that 2D-MPOD and OFA may provide effective biomarkers.

## Introduction

Age-related macular degeneration (AMD) continues to be a leading cause of irreversible central blindness and visual impairment worldwide [1, 2] and poses a significant public health burden [3]. AMD is a vascular-metabolic-inflammatory disease [4]. The dual origins of lipoproteins-fatty acids and cholesterol and photoreceptor outer segments form soft drusen and basal linear deposits (reticular pseudodrusen). They are the major risk factors for end-stage AMD [5], exudative AMD (eAMD), or geographic atrophy (GA) [6]. Intermediate AMD (iAMD) defined mainly by medium to large drusen, as distinct from normal aging with small soft drusen, also termed as drupelets [7], along with the floor of microangiopathy is accelerated further to develop late-stage AMD [8–11] and is now recognised as a target for future interventions. We therefore need tools for future clinical endpoints which accurately characterise iAMD.

Photo-oxidative retinal injury is thought to play a role in AMD. The macular pigments (MPs) such as lutein, zeaxanthin, and meso-zeaxanthin are mainly concentrated in the fovea and filter damaging blue light [12–14]. Reduced MP optical density (MPOD) is a risk factor for AMD [15, 16]. Methods for measuring MPOD include heterochromatic flicker [17], reflectometry [18], autofluorescence [19], and Raman spectroscopy [20] (for a review see [21]). Generally, the average MPOD within a part of the central retina is measured. The Heidelberg Spectralis OCT (Heidelberg Engineering GmbH, Germany) provides an autofluorescence method, the raw data for which is 2-dimensional (2D) MPOD data spanning the whole macula. The device software reports a 1-dimensional (1D) radial average computed around the polar angles out to a radius of 6 to 9°. Various studies of AMD have examined averages across that 1D function to yield a single aggregate MPOD value, which has good reproducibility [22] but relatively poor diagnostic value [23–26].

Thus far, few studies have attempted to correlate physiological measures of 2D macular function with the 2D distribution of MPOD in AMD. One study compared MPOD data with several measures including the mean of sensitivities to 5 Goldmann Size III (GS3) stimuli presented within the central 2° and scotopic sensitivity to a 3° wide target at 5° eccentricity [25]. We have published 5 studies of multifocal pupillographic objective perimetry (mfPOP) showing good diagnostic power in early- to intermediate-stage AMD [27–31]. All of those studies included an mfPOP variant called P131 that tests 44 regions within the central ± 15° of the visual field. Both eyes are tested concurrently for 7 min, and both sensitivity and response delay are reported at each region. An objective of this study is to compare these measures of retinal function with 2D-MPOD data taken from retinal regions that exactly match 20 mfPOP stimulus locations/macula in eyes with early to late-stage AMD. We also examine changes in 2D-MPOD over the 1-year study period and the diagnostic power for discriminating early- from later-stage AMD of mfPOP, MPOD, and their pairwise combinations and compare those with outcomes from standard perimetry and visual acuity.

## Methods

### Subjects

In this prospective study, an FDA-cleared prototype of the mfPOP, the ObjectiveFIELD Analyser (OFA) device (Konan Medical USA, Irvine, CA), was used to present stimuli and monitor the pupillary diameter. A total of 29 subjects completed both OFA and MPOD testing in two test sessions. The pairs of sessions were separated by 0.99 ± 0.16 years. Eyes were graded to the 4-step Age-Related Eye Disease Study (AREDS) standards [32], where grades 2 and 3 closely correspond to iAMD [7]. Stage AREDS-4 is characterised by either eAMD or GA. We excluded persons who had ophthalmological or neurological diseases other than AMD that would potentially affect visual acuity, visual fields, or pupillary function: lens or media opacities, trauma to eyes or head, and systemic diseases such as diabetes and hypertension. This report covers the findings of only the preliminary study with a short follow-up period and a cohort of patients who participated in the early phase of the study. Thus, it has a random number of participants and eyes.

This study was approved by the Australian Capital Territory Health Human Research Ethics Committee (ETH 7.07.667) and Australian National University Human Ethics Committee (2010/194), and informed written consent was obtained. The research adhered to the tenets of the Declaration of Helsinki.

### Ophthalmic examinations

We performed 10–2 visual field testing with the Matrix perimeter (Carl Zeiss Meditec Inc., Dublin, CA), best-corrected visual acuity (BCVA), and low contrast BCVA (lowC VA) (both using log-MAR charts), slit-lamp examination to rule out pupillary abnormality and media opacity, and 90-dioptre bio-microscopy for retinal evaluation, measured intraocular pressure (IOP) with Goldmann applanation tonometry, pachymetry (Pachmate DGH 55, DGH Technology Inc., USA), and corneal curvature with auto-refractometer (ARK-1 s NIDEK co. Ltd, AICHI Japan). The pupils were dilated with 1% tropicamide eye drops. Macular photographs, normal and × 2 magnified, were taken with a fundus camera for AMD grading (CR-2 Digital Retinal Camera, Canon Inc., Tokyo, Japan). Those colour photographs were used for AREDS grading [32] by two retinal specialists independently. Macular retinal thickness posterior pole scans providing both 8 × 8 grid data and 9 ETDRS subfield data with 25 ART frames, 61 sections, and line spacing of 120 µm, retinal nerve fibre layer (RNFL), and MPOD imaging were performed with a Spectralis OCT (Heidelberg Engineering GmbH, Germany).

The diagnosis and grading of the AMD were performed by two retinal specialist ophthalmologists (authors REW and BBR) independently. Any disagreement was given the final verdict by the third retinal specialist (author RCB). The clinical tests and imaging were performed by an optometrist (author FS), an orthoptist (author EMFR), and an ophthalmologist (author BBR). The analysis was done by a mathematician (author JPvK) and a senior vision scientist (author TM).

### Multifocal pupillographic objective perimetry

**Fig. 1:**
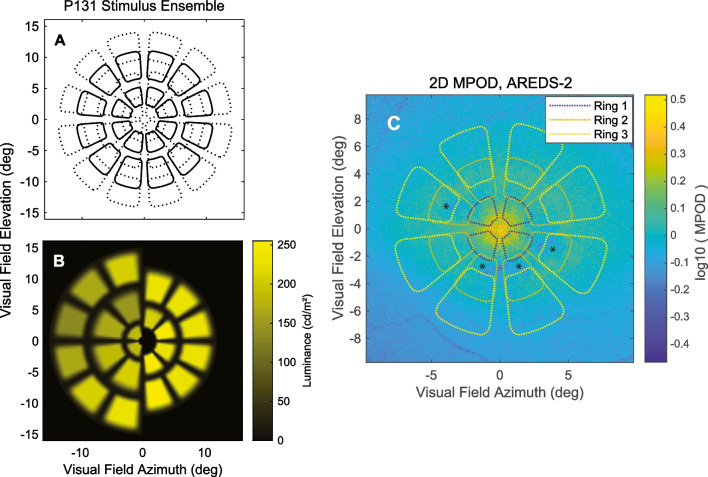
Representations of the ObjectiveFIELD Analyser (OFA) stimuli and 2-dimensional macular pigment optical density (2D-MPOD). **A** Contours of the 44 regions of P131. In practice, they are never presented overlapping. **B** The left and right halves of the 5 rings of P131 stimuli showing their relative intensities. The central 6 mm of the retina corresponds to the central 20° of the visual field. Therefore, the inner three rings of **A**, 20 stimuli, are within the region where the 2D-MPOD signal is measured (± 18°). The stimuli of the central three rings had areas of 6.87, 12.6, and 28.3°, centred on 1.75, 4.0, and 6.0° eccentricity. **C** Example of a 2D-MPOD image. The calibration bar at right shows the relative optic density in log10 units relative to the 9° reference eccentricity. Thus, 0 means 1 × the reference level, − 0.3 represents 0.50 × of that level, and 0.4 is 2.51 × . There is thus a fivefold difference in optical density across the image. The image is that of a left eye projected into visual space. The optic disc is therefore on the left. The lozenge-shaped regions (dashed lines) represent the borders of the 20 central-most of the OFA stimuli of **A**. The eye was classified as AREDS-2. The * indicates areas with patches of low MPOD − 0.3 to − 0.4 log-units corresponding to darker blues

An issue with GS3 stimuli is that they stimulate < 0.4% of the area of standard perimetry contributing to their poor reproducibility [33, 34]. In the macular 10–2 test pattern, the GS3 stimuli stimulate < 3.4% of the test grid area. According to sampling theory, much larger stimuli that substantially test the whole field are needed [33]. The OFA stimuli used here meet those criteria (Fig. 1). OFA was performed before any other eye tests. The participants did not drink caffeinated beverages within an hour of the test. OFA concurrently presents independent multifocal stimuli to both eyes while measuring direct and consensual responses from every visual field location stimulated [35]. Any vergence deficit was corrected before starting the tests. The maximal luminance of the stimuli was 288 cd/m^2^ presented on a 10 cd/m^2^ background (Fig. 1A, 1).

OFA and other analyses employed MATLAB (2020b, The MathWorks, Natick, MA). The response waveforms for each of the 44 test OFA regions per eye were extracted from raw pupillary responses using a multiple regression method [36]. The average response waveforms of each retinal region were fitted to a log–normal function [37, 38], allowing per-region constriction amplitude (sensitivity) and time-to-peak (delay) to be measured. The regression method also generated standard errors for all the estimated sensitivity and delays [35, 39].

### Macular pigment optical density

**Fig. 2:**
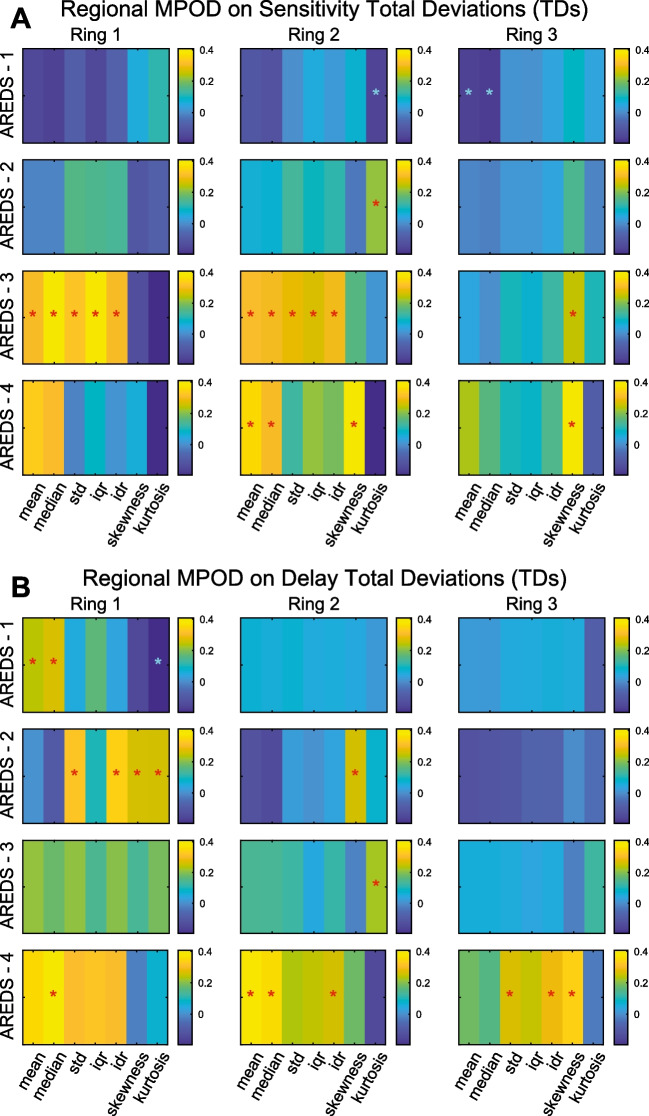
Summary of correlations between 2-dimensional macular pigment optical density (2D-MPOD) data plotted on ObjectiveFIELD Analyser (OFA) total deviations (TDs). The red ‘*’ indicates significant correlation of the typical sign (see text) and cyan ‘*’ the opposite sign. **A** Comparisons with OFA sensitivities, **B** OFA delays. The colour calibration scales indicate the degree of correlation. MPOD = macular pigment optical density; IDR = inter-decile range; IQR = inter-quartile range; STD = standard deviation

Two Spectralis OCT devices were used. This meant that the fluorescence images were taken with exciting green and blue lights at either 514.1 nm and 484 nm or 518.6 nm and 486.8 nm respectively. The appropriate extinction coefficients of 0.253 and 0.793 or 0.173 and 0.783 were used to calculate MPOD [19, 23, 24]. The peripheral reference eccentricity was set to 9° to match the outer border of the central 20 P131 stimuli. That reference eccentricity has been used in recent MPOD studies [23, 25, 40]. The raw autofluorescence images were extracted from PNG files to generate MPOD images (Fig. 2) [24]. We computed the 1D polar-angle averages to confirm that they were a good match to the standard Spectralis OCT 1D MPOD reports. We then cookie-cuttered out regions from the MPOD images corresponding to the central 20 P131 stimuli at 10% of their maximum luminance (e.g. Figure 1C). These stimuli comprised 3 rings.

For each of the resulting lozenge-shaped 2D-MPOD regions/eye, we computed seven measures on the pixel values within each region: the mean, median, standard deviation, skewness, kurtosis, inter-quartile range (iqr), and inter-decile range (idr). The Bland–Altman plots [41] comparing the data obtained from the first and second sessions 0.99 ± 0.16 years apart were examined. These are plots of the differences at each region between the two test visits, plotted on the means from the visits. To examine any change over the study period, linear models fitting the mean difference and slope (change over time) as a function of the mean differences were fitted to the data. The P131 test described here is now known as the OFA15 test on the OFA device.

### Statistics and receiver operator curve analysis

As no lab had investigated regional MPOD, there was no data for power calculations. We have published standardised effect sizes for P131 delays [42]. For our AREDS-2 and AREDS-3 subjects, the effect size is 1.28. Power calculation used G*Power (Version 3.1.9.7, University of Kiel, Germany) and indicated for *p* = 0.02 and power of 0.98, and the group size was 22. This was deemed adequate to proceed with this pilot study.

For the receiver operating characteristic curve (ROC) analysis, we report the percentage area under ROC plots (%AUROC). We examined correlations between the OFA per-region sensitivities and delays and the seven MPOD measures. For all of those, we calculated the standard perimetry measures: total deviations (TDs) and pattern deviations (PDs). The TDs are per-region deviations from control data, and the PDs are computed from the TDs. We also examined asymmetries between the two eyes. We opted for a conservative control data set which was the median at each region across subjects and eyes. Note that the right-eye data was first flipped left to right so that all data was left-eye equivalent. Our control group was the 20 eyes from subjects in which both eyes were AREDS-1. These eyes are often considered to be clinically normal [7]. However, P131 has been reported to discriminate normal control eyes from AREDS-1 eyes with a %AUROC of about 85% [28].

Given that the AREDS severity was frequently different between eyes, the ROC analysis was performed by eye rather than by subject. Hence, we looked at the ability to diagnose different AREDS steps from control eyes. For the OFA data, we used our standard ROC analysis method [39] of recomputing the %AUC for means of selections of the N worst (most deviating from normal) regions to obtain an indication of the number of diagnostic regions appearing in the measured visual field data. Scores from linear combinations of pairs of variables [39] were also examined. For some variables such as BCVA, Matrix perimeter summary statistics, MD, and PSD, we calculated the standardised effect sizes for discriminating data from pairs of AREDS severities as Hedge’s *g* and converted those to %AUROC [43].

## Results

### Demographics

A total of 29 subjects (71.6 ± 6.3 years, 22 females, mean ± SD) completed two test sessions of OFA and MPOD testing 0.99 ± 0.16 years apart. Among the 58 eyes, the distribution of eyes per AREDS step was AREDS-1, 26; AREDS-2, 15; AREDS-3, 10; and AREDS-4, 7. The AREDS-4 eyes all had exudative AMD. Fourteen subjects had differences in AREDS steps between the eyes of 0, and 15 had differences of 1.

Table 1 summarises some standard eye measurement data by AREDS severity level. The acuities are in letters. The rightmost three columns indicate AUROC values where AUC_1,*n*_ indicates that AREDS levels 1 and *n* (range *n* = 2 to 4) are being compared. When comparing AREDS-1 with AREDS-4 eyes, the Matrix PSD provided the best discrimination at 83.4%.

**Table 1:** Visual characteristics of eyes by AREDS severity

Measure	AREDS-1	AREDS-2	AREDS-3	AREDS-4	AUC_1,2_	AUC_1,3_	AUC_1,4_
BCVA	80.4 ± 15.7	86.0 ± 4.23	86.0 ± 7.62	74.0 ± 11.7	63.3%	50.8%	62.7%
lowC VA	71.5 ± 14.5	75.5 ± 4.96	86.0 ± 20.6	59.4 ± 11.4	60.0%	75.2%	74.0%
Matrix MD	0.03 ± 2.91	0.19 ± 2.22	− 1.37 ± 2.35	− 2.25 ± 3.31	51.8%	62.4%	69.5%
Matrix_PSD	3.02 ± 0.59	2.93 ± 0.32	3.71 ± 0.98	4.18 ± 1.03	55.0%	71.7%	83.4%

*AREDS* Age-Related Eye Disease Study [32], *BCVA* best-corrected visual acuity in letters, *lowC VA* low contrast visual acuity, *MD* mean defect, *PSD* pattern standard deviation, *Hg* Hedge’s *g*

### Correlations between OFA and MPOD

We computed Spearman’s correlations between the 20 per-region MPOD data and the OFA total deviations (TDs) at the same regions for each of the 3 rings and the 4 AREDS severities. Generally, the correlations between MPOD measures and OFA sensitivities were positive. Thus, lower sensitivities were associated with lower MPOD values. Per-region delays mainly produced negative correlations such that lower MPOD was associated with longer delays or greater variability. Given the 20 regions/macula, we performed a Bonferroni correction for those multiple comparisons. After correction, correlations with a magnitude exceeding ± 0.25 were consistently significant (*p* < 0.05).

Figure 2 summarises the MPOD correlations with OFA sensitivities (Fig. 2A) and delays (Fig. 2B). A red ‘*’ indicates a correlation with the typical sign (+ ve for sensitivities, − ve for delays), and the cyan ‘*’ indicates the significant correlation had the non-typical sign. For sensitivities, most significant correlations were associated with MPOD variability measures within the 2D regions of rings 1 and 2 (std, iqr, idr). Central tendency measures, mean and median MPOD, were significantly correlated with AREDS 4, Ring 2. For delays, the variability measures were significantly correlated with AREDS-2 and AREDS-3 in Ring 1 but also for AREDS-4 in the peripheral Ring 3. Summary statistics based on higher-order moments, the skewness and kurtosis, were rarely significant. When kurtosis was significant, it tended to adopt the non-typical sign such that high kurtosis (long-tailed distributions) was associated with low sensitivity and long delays.

### Change of MPOD over time

**Fig. 3:**
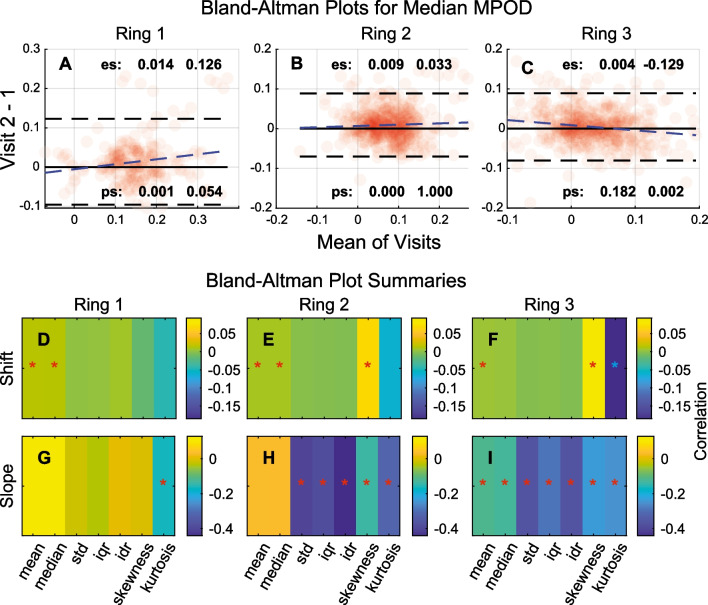
Two-dimensional macular pigment optical density (2D-MPOD) change over time. **A**–**C** Bland–Altman plots for the median pixel values in each region. The points are translucent dots so that the density of the distributions can be appreciated. The fitted model is shown as a blue dashed line. The pairs of values labelled ‘es’ are the estimated between-visit shift and slope. The two values labelled ‘ps’ are the respective *p*-values. The horizontal black dashed lines are the 95% confidence limits. The horizontal black line marks difference. A ‘*’ indicates significant values. **D**–**F** The fitted vertical shift values. **G**–**I** Fitted slopes. MPOD = macular pigment optical density; IDR = inter-decile range; IQR = inter-quartile range; STD = standard deviation

Each person was tested twice about 1 year apart (the “Methods” section), and a Bland–Altman analysis was performed to investigate changes in the measures over time. Aside from the standard plots of between-visit differences on between-visit means (Fig. 3A–C), we also fitted a linear model investigating the between-visit vertical shift and slope (change over time, blue dashed lines). Figure 3A–C shows examples of the Bland–Altman plots for the median MPOD for Rings 1 to 3. Summary plots of the fitted offsets are shown in Fig. 3D–F and the slopes in F3g. 3G–I. As in Fig. 3, the *p*-values were Bonferroni-corrected, and significant estimated values are tagged with a ‘*’. Except for kurtosis the significant vertical shifts were all positive, i.e. the values increased significantly on the second visit independent of the mean between-visit value. Those shifts were relatively small, however: 0.030 ± 0.035 (mean ± SD). The significant slopes were all negative and reasonably large: − 0.277 ± 0.034; hence, initially large values on visit 1 became smaller on visit 2. Interestingly, most of the significant slopes were in Rings 2 and 3. The slopes for the mean and median were significant in Ring 3 (F3g. 3I).

### OFA and 2D-MPOD diagnostic power

We assessed the diagnostic power (%AUROC) of both the OFA data from the central 20 regions of P131 and the matching 2D-MPOD data types. Diagnostic power was quantified as a percentage area under receiver operator characteristic plots (%AUROC). We also examined scores derived from pairwise linear combinations of OFA and MPOD measures [39]. We found that total deviations provided more informative data than pattern deviations and asymmetries so we do not report the latter two.

The diagnostic performance is summarised in Table 2, which shows %AUROC ± SE. As mentioned in the “Methods” section, we sorted the deviations from control data and then repeated the ROC analysis using the mean of the worst N regions, beginning with the single worst, and ending with the mean of all 20 regions. This meant that each cycle of ROC analysis was based on one number for each eye. The single worst point in every field was excluded. For simplicity, we report on the data for the mean of the 10 worst points in Table 2. Sometimes, better performance was obtained for smaller or larger *N*, but 10 is representative.

**Table 2:** Diagnostic power between AREDS-1 eyes* and more severe eyes

Measure	AREDS-2	AREDS-3	AREDS-4
2D-MPOD median	47.6 ± 7.1	80.0 ± 6.3	85.4 ± 6.2
2D-MPOD IDR	34.4 ± 6.7	52.0 ± 9.6	69.6 ± 9.1
2D-MPOD Kurtosis	44.3 ± 6.9	58.5 ± 7.7	76.8 ± 6.8
2D-MPOD media + IDR	52.5 ± 7.1	83.5 ± 5.8	87.3 ± 5.3
OFA sensitivity central 20	53.1 ± 7.0	74.1 ± 6.8	76.8 ± 7.8
OFA sensitivity all 44	57.4 ± 7.0	76.4 ± 6.6	77.3 ± 7.0

*AREDS* Age-Related Eye Disease Study [32], *2D-MPOD* 2-dimensional macular pigment optical density, *IDR* inter-decile range, *OFA* ObjectiveFIELD Analyser

^*^The control group was 20 eyes from persons where both eyes were AREDS-1. All values are %AUROCs

The best-performing 2D-MPOD measures were the mean and median of the per-region pixel data. These results were nearly identical, so we report on the median MPOD in Table 2. Of the measures reporting on 2D-MPOD variability (std, iqr, idr), the inter-decile range was marginally the best. Of the higher-order statistics, negative Kurtosis performed better than Skewness. The fact that negative kurtosis performed best meant that regions with greater AREDS severity had larger kurtosis, i.e. their within-region pixel distributions had broader tails (leptokurtotic). This agreed with correlations with OFA data. We also examined linear combinations of paired measures (the “Methods” section). The best combination was two 2D-MPOD measures: the medians and the inter-decile ranges (Table 2, Median + IDR). We also examined the OFA sensitivities, both for the central 20 regions and all 44 regions (Table 2, sensitivities 20 and 44 respectively). OFA delays performed less well.

## Discussion

It is well known that retinal changes due to AMD can precede changes in BCVA, especially changes measured by mfPOP [31]. Some other functional vision tests, including blue-cone function, dark adaptation, and flicker perimetry, largely only detect late-stage AMD (reviewed [28]). Therefore, new measures to provide clinical endpoints for iAMD are needed.

Spearman’s correlations were selected here because the delay data tended to show slightly curvilinear relationships. For OFA sensitivities, the significant correlations with 2D-MPOD data were predominantly in Rings 1 and 2 of AREDS-3 and AREDS-4. These included both measures of central tendency and variability. OFA delays provided significant correlation in Ring 1, AREDS-2 and all rings of AREDS-4. Taken together, this cross-sectional data might suggest some ability of 2D-MPOD data to provide prognostic information. Previous studies with P131 in early-stage exudative AMD indicated that differences between central and peripheral macula were either indicative of response to initial treatment with anti-VEGF [44] or were predictive of the need for anti-VEGF treatment [30] on *prn* management over 15 months. Here, significant negative shifts in mean and/or median MPOD were observed in all rings over the course of the study (Fig. 3D–F) and also in the temporal slopes of measures associated with MPOD variability in Rings 2 and 3 (Fig. 3H, 3).

Aside from significant correlations with OFA measures and change over the course of a year, some 2D-MPOD measures showed reasonable ability to discriminate AREDS-3 and AREDS-4 eyes from AREDS-1 (Table 2). An %AUROC of 80.0 ± 6.29% was achieved by the MPOD per-region medians. The combination of those medians and the inter-decile ranges increased that to 83.5 ± 5.8. This suggests that 2D-MPOD measures might be suitable for clinical endpoints in studies of interventions in iAMD over time periods of about 1 year. An important point here is that the ROC analysis used sorted deviations from control data, strictly means of the worst N regions. Thus, the ROC models did not depend on the location of damage. Thus, the damage appeared to be patchy within the 2D sampling array. Information about such 2D patchiness will be largely lost in the conventional polar-angle means that are frequently reported [23–26]. Overall, this indicates that 2D analysis is worth pursuing. MPOD measured within small regions has been reported to not be correlated with rod adaptation in older subjects without AMD [45].

AMD impacts both developed and developing countries with more than 170 million individuals affected globally [1]. In developed countries, AMD affects nearly 10% of those people older than 65 years and 25% of those older than 75 years [46]. In the USA, the total economic impact of late-stage AMD was estimated at USD 49.1 billion in 2022 [47]. In a small developing country like Bhutan, AMD was the fourth most common retinal disorder [48], with 43.6% of those patients being late-stage at their first presentation [49]. eAMD was the most common (42.2%) indication for intravitreal anti-VEGF injection [50]. Similarly, in Nepal, AMD was the most common retinal disorder among patients attending vitreoretinal clinics in tertiary eye care centres [51]. While the developed and populous countries are burdened to manage a large number of AMD cases [47], the developing countries are challenged with limited human resources and accessibility to standard healthcare services [48]. Diagnostic approach such as 2D-MPOD with high diagnostic power capable of identifying at-risk eyes, or clinching diagnosis of early-stage AMD, would be beneficial to both the developed and developing countries. It can potentially reduce blindness and visual impairment, visual disability, and economic impact.

This preliminary study is limited by a small number of patients and a short follow-up period. The control group that we considered was the 20 eyes from the subjects in which both eyes had AREDS-1 disease. A larger control group and true normal eyes would have been desirable. In comparison with normal controls, these eyes are well discriminated by OFA [28].

In conclusion, significant correlations between OFA sensitivities and delays and 2D-MPOD were observed, and these were related to AMD severity indicating a reasonable structure/function relationship. Significant changes in 2D-MPOD were observed over a year, and these were also related to disease severity. Several 2D-MPOD measures produced good diagnostic power for discriminating AREDS-1 eyes from later-stage AMD which may indicate power to track independent aspects of iAMD. Even when using a very simple normative model, the ability of 2D-MPOD to distinguish AREDS-1 and AREDS-3 was good. Overall, the results indicate that further investigation of 2D-MPOD to provide biomarkers for AMD is warranted.

## Data Availability

The data and material are available from the corresponding author.
